# Parasitic myoma after transabdominal hysterectomy for fibroids: a case report

**DOI:** 10.1186/s12905-023-02410-3

**Published:** 2023-06-16

**Authors:** Jiao Wang, Guipeng Liu, Qing Yang

**Affiliations:** Department of Obstetrics and Gynecology, Shengjing Hospital of China Medical University, No.36 Sanhao Street, Heping District, Shenyang, Liaoning Province 110004 China

**Keywords:** Parasitic myoma, Transabdominal hysterectomy, Morcellation

## Abstract

**Background:**

Parasitic myomas typically occur after a pedunculated subserosal fibroid loses its uterine blood supply and parasitizes other organs or after a surgery involving morcellation techniques. Parasitic myomas that occur after transabdominal surgery are extremely rare and may not be sufficiently documented. Here, we present a case of parasitic myoma in the anterior abdominal wall following a transabdominal hysterectomy for fibroids.

**Case presentation:**

The patient was a 46-year-old Chinese woman who had undergone surgery for uterine myomas at our hospital 1 year prior. The patient later revisited our department with a palpable mass in her abdomen, and imaging revealed a mass in the iliac fossa. The possibility of a broad ligament myoma or solid ovarian tumor was considered before surgery, and laparoscopic exploration was performed under general anesthesia. A tumor measuring approximately 4.5 × 4.0 cm was found in the right anterior abdominal wall, and a parasitic myoma was considered. The tumor was completely resected. Pathological analysis of the surgical specimens suggested leiomyoma. The patient recovered well and was discharged on postoperative day 3.

**Conclusion:**

This case suggests that parasitic myoma should be considered in the differential diagnosis of patients presenting with abdominal or pelvic solid tumors with a history of surgery for uterine leiomyomas, even without a history of laparoscopic surgery using a power morcellator. Thorough inspection and washing of the abdominopelvic cavity at the end of surgery is vital.

## Background

Parasitic leiomyomas are rare extrauterine smooth muscle neoplasms [1]. They typically occur on two occasions: (1) after a pedunculated subserosal fibroid loses its uterine blood supply and survives by obtaining blood supply from other neighboring organs or (2) after a surgery that can produce fibroid fragments, particularly one that involves morcellation techniques [2, 3]. Owing to the increase in laparoscopic surgery and the use of power morcellators, several cases of parasitic myomas related to this technique have been reported and have attracted the attention of gynecologists. However, parasitic myomas that occur after transabdominal hysterectomy are extremely rare and may not be sufficiently documented. Herein, we present a case of parasitic myoma located in the anterior abdominal wall following a transabdominal hysterectomy for fibroids.

## Case presentation

**Fig. 1:**
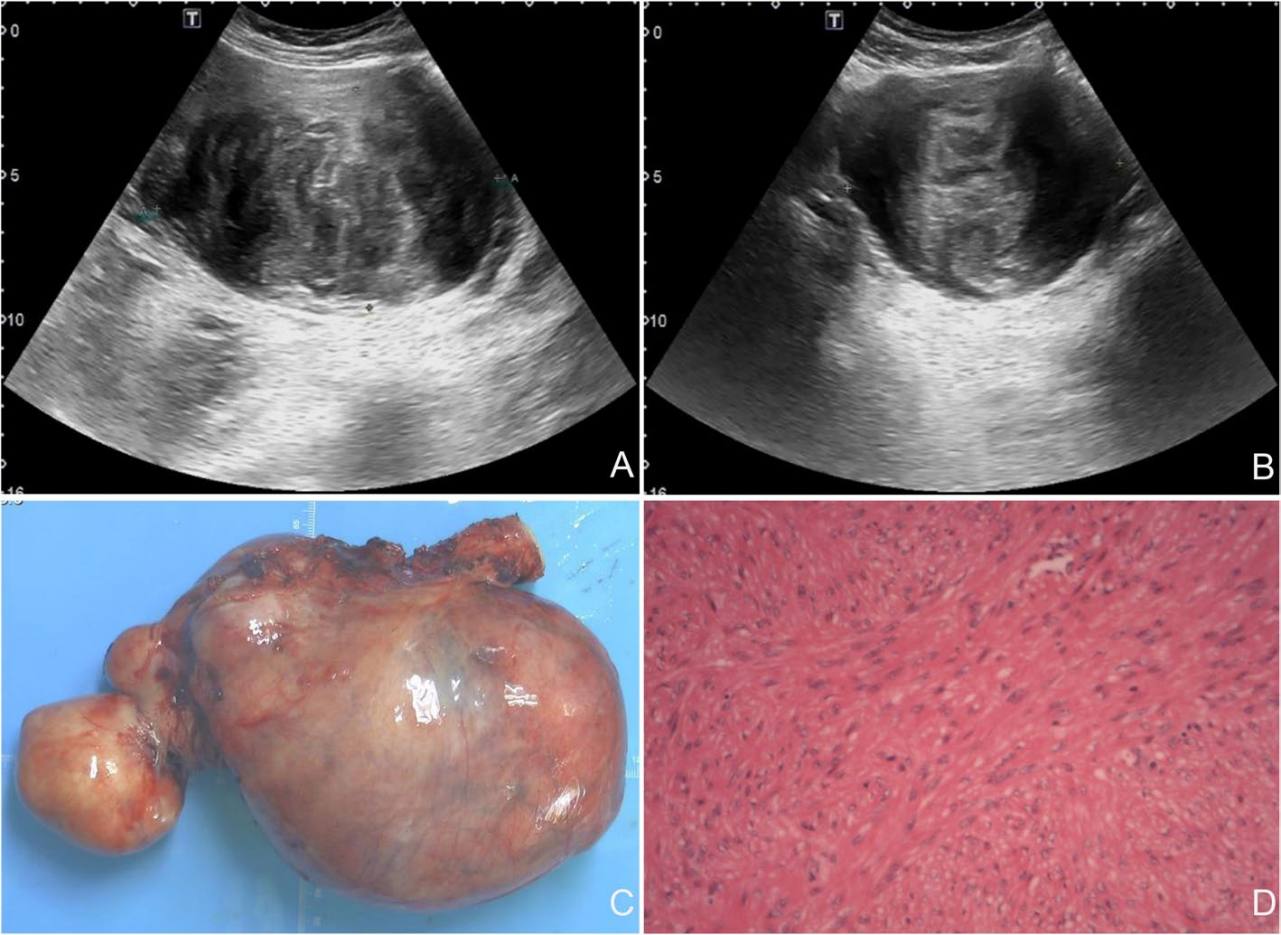
Imaging and histopathological findings during the patient's first hospitalization. **A**, **B** Pelvic ultrasound revealed multiple hypoechoic masses in the uterus, the largest of which was approximately 12.9 × 10.4 × 7.6 cm; **C** The gross pathology of the uterus revealed multiple fibroids; **D** Microscopic examination revealed spindle-shaped tumor cells arranged in a braided pattern (H&E staining, × 200)

The patient was a 46-year-old Chinese woman (gravida 1, para 1). One year prior to presentation, she visited our hospital for surgery to remove uterine myomas identified on pelvic ultrasound. Multiple hypoechoic masses in the uterus, the largest of which was approximately 12.9 × 10.4 × 7.6 cm (Fig. 1A, B), and a compressed endometrium were observed on pelvic ultrasound. Therefore, a total transabdominal hysterectomy and bilateral salpingectomy were performed through the transverse incision of a previous cesarean section.

During the operation, multiple myomas were observed in the uterus, the largest of which (12 × 10 cm) was located in the right anterior wall (Fig. 1C). The operation was uneventful, and there was no evidence of myomas in any extrauterine location at the time of surgery. Paraffin-embedded pathology revealed multiple uterine leiomyomas. Microscopic examination revealed spindle-shaped tumor cells arranged in a braided pattern (Fig. 1D). No abnormalities were found in the outpatient review at 1 month postoperatively.

**Fig. 2:**
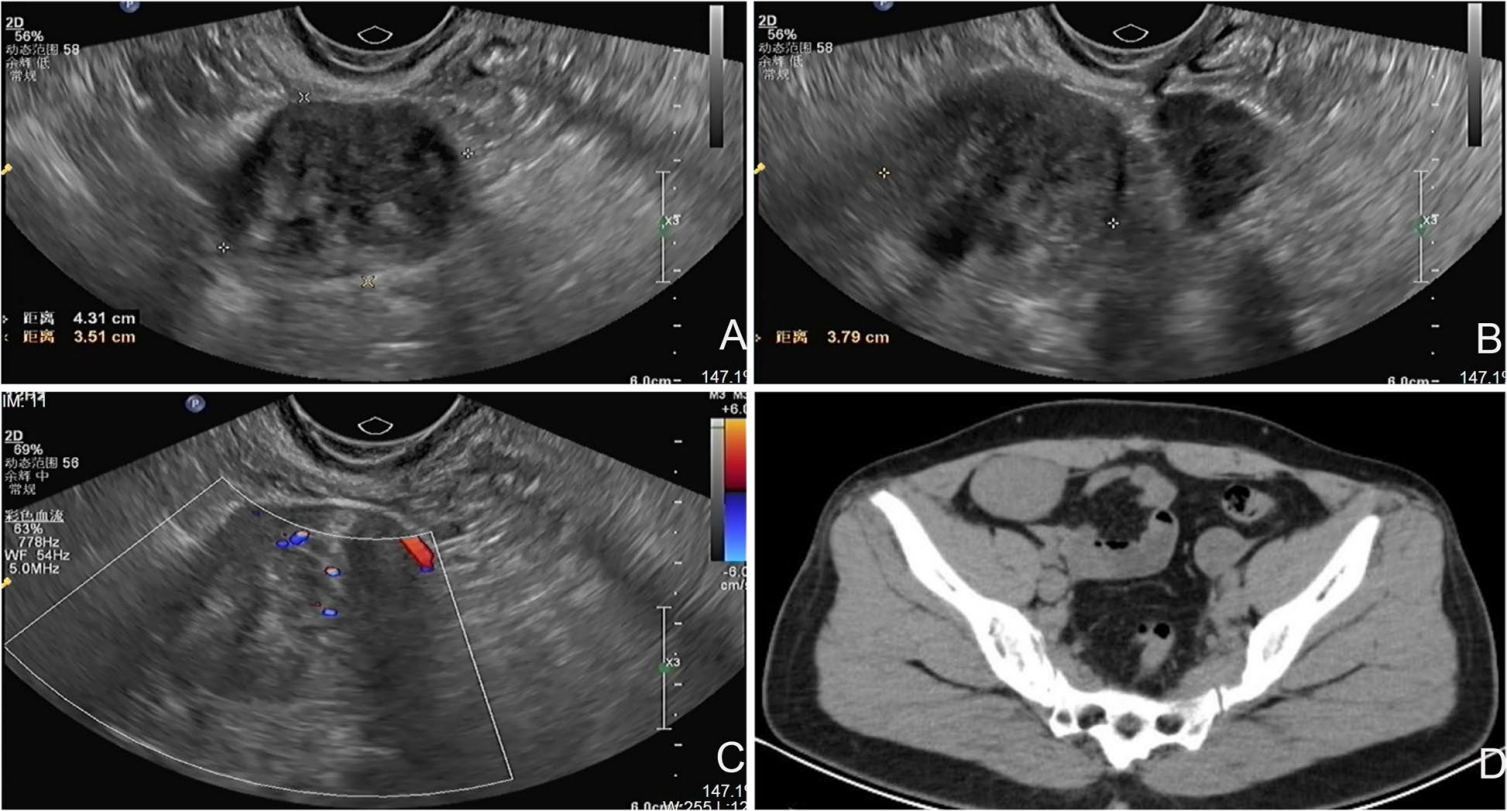
Imaging findings of the patient during the second hospitalization. **A**, **B** Pelvic ultrasound revealed a 4.3 × 3.8 × 3.5 cm mass in the right iliac fossa with a clear boundary and heterogeneous medium and low echo; **C** Color Doppler flow imaging revealed blood flow signals; **D** Abdominal CT revealed an 4.6 × 3.1 cm oval soft tissue density mass in the right iliac fossa. The CT value was approximately 53 Hounsfield units, the boundary was clear

Twenty days ago, the patient revisited our department with a 4 cm palpable mass in the right lower quadrant of the abdomen. Pelvic ultrasound revealed a 4.3 × 3.8 × 3.5 cm mass in the right iliac fossa with a clear boundary and heterogeneous medium and low echo (Fig. 2A, B). Color Doppler flow imaging revealed blood flow signals (Fig. 2C). Abdominal computed tomography (CT) revealed an oval, soft tissue density mass in the right iliac fossa. The CT value was approximately 53 Hounsfield units, the boundary was clear, and the size was 4.6 × 3.1 cm (Fig. 2D). The serum carbohydrate antigen-125 level was normal. A broad ligament myoma or solid ovarian tumor was considered before surgery, and laparoscopic exploration was performed under general anesthesia.

**Fig. 3:**
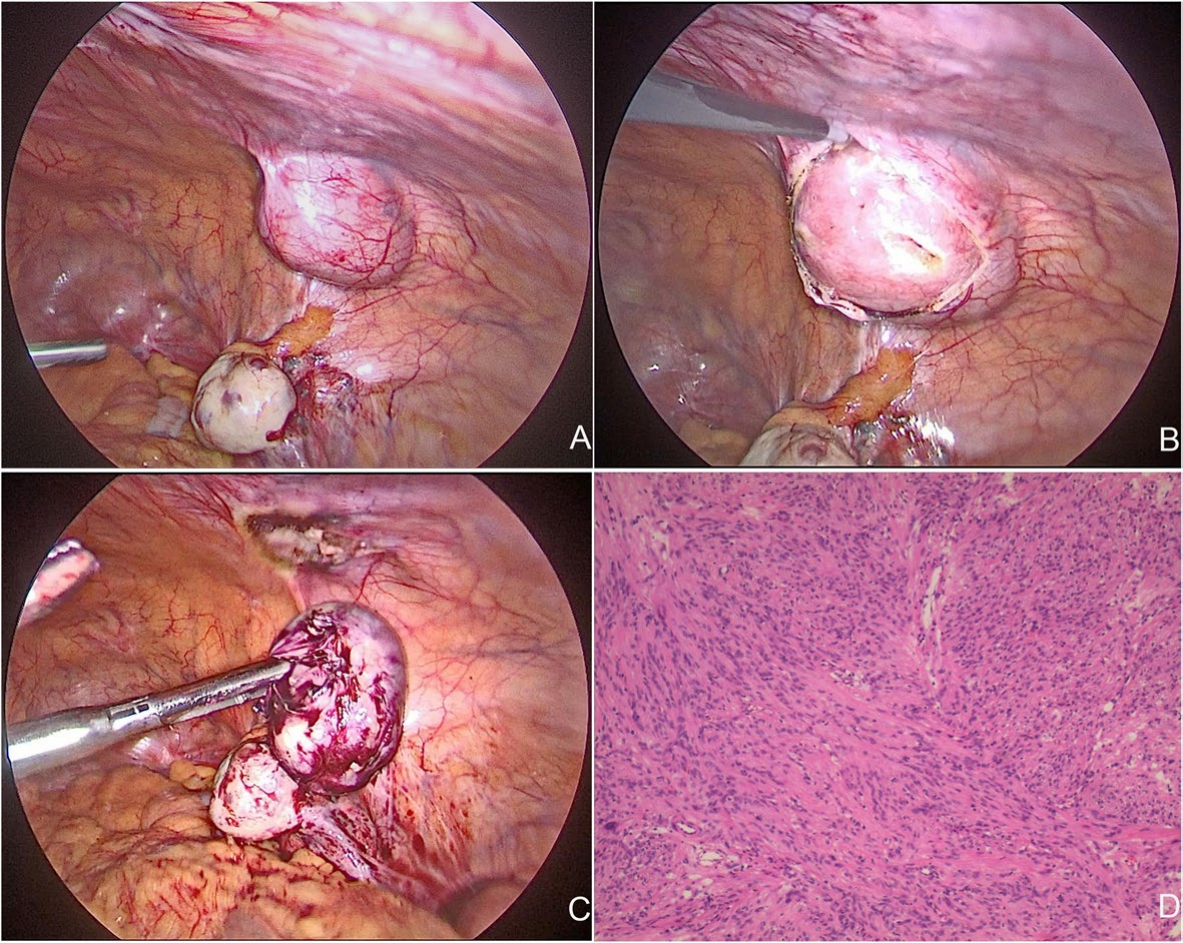
Laparoscopic and histopathological findings of the patient during the second hospitalization. **A**, **B**, **C** Laparoscopic exploration revealed a 4.5 × 4.0 cm tumor with a smooth surface could be seen in the right anterior abdominal wall. The color was pinkish white; **D** Microscopic examination revealed spindle-shaped tumor cells arranged in a braided pattern (H&E staining, × 100)

During the operation, a tumor measuring approximately 4.5 × 4.0 cm could be seen in the right anterior abdominal wall, which is located approximately 3 cm from the right edge of the previous transverse incision. The color was pinkish white and the surface was smooth (Fig. 3A, B, C); a parasitic myoma was considered. The uterus and bilateral fallopian tubes were absent and both ovaries were normal. The peritoneum on the surface of the tumor was incised using a monopolar electric hook, and the tumor was completely resected. The tumor was placed in a retrieval bag and removed from the abdominal cavity. Specimens were sent for frozen pathology analysis; the results were benign. Paraffin-embedded pathological results suggested leiomyoma. Microscopic examination revealed spindle-shaped tumor cells arranged in a braided pattern (Fig. 3D). The patient recovered well and was discharged on postoperative day 3. No abnormalities were found during the outpatient follow-up at 1 month postoperatively.

## Discussion

Uterine myomas are the most common benign tumors of the female reproductive system. A variety of treatment modalities including both medical and surgical interventions have been used in symptomatic patients [4]. However, parasitic myomas, which are defined as leiomyomas that are not attached to the uterus and are “parasitic” because they receive their blood supply from the surrounding organs [5], are not common in the clinic. Parasitic myomas may occur spontaneously as pedunculated subserosal myomas lose their uterine blood supply and parasitize other organs [2, 6] or may be iatrogenically created after surgery, particularly one that uses morcellation techniques [2]. The reported incidence of parasitic myomas after laparoscopic morcellation is 0.12–0.95% [5, 7–9]. Even with correct use and careful observation, the morcellator may produce minute fibroid tissue, and the residual fibroid fragments after morcellation can be implanted onto normal tissue anywhere in the abdominal cavity [10].

Our patient had undergone a transabdominal total hysterectomy for multiple and large uterine leiomyomas 1 year prior. No morcellator was used during the first surgery. Therefore, we considered that the solid mass was a broad ligament myoma or a solid tumor derived from the right ovary. However, the final diagnosis was parasitic leiomyoma. We think that this parasitic myoma may have been formed because of the implantation of tiny leiomyoma fragments unintentionally generated during her first operation.

The clinical manifestations of this condition are non-specific and correlate with the location and size of the parasitic myoma. The most common locations of parasitic myomas are the entry points of laparoscopic surgery, the broad ligament of the uterus, abdominal peritoneum, pouch of Douglas, sigmoid colon, and greater omentum; these are areas with good blood supply [11]. Parasitic myoma may be asymptomatic and may be found only incidentally during examination [12] or surgery [8]. Additionally, it can present as a palpable mass [4, 13], similar to that observed in our patient; as compression symptoms of the corresponding organs, such as frequent urination [11], constipation [11], abdominal distension [9], and hydronephrosis [14]; as abdominal and pelvic pain [3, 15–17]; or as dyspareunia [8].

The diagnosis of parasitic myoma is challenging because of its rarity and atypical presentation. Diagnosis should be combined with consideration of medical history, physical examination, ultrasound, and magnetic resonance imaging (MRI) or CT, and the final diagnosis should be based on histopathology. Ultrasound is the standard initial investigation tool, and a typical leiomyoma generally has a whorled appearance with echogenicity similar to that of the myometrium, but sometimes may be hypoechoic or with variable echogenicity [18]. Pelvic MRI is a highly accurate technique for demonstrating uterine leiomyomas. The classic appearance of a typical fibroid on MR is that of a well-circumscribed mass with homogeneous T2 hypointensity and T1 isointensity relative to the myometrium [18].

Management generally involves surgical resection, which can be performed using laparotomy, laparoscopic, or robotic procedures [11, 19]. A thorough inspection and washing of the abdominopelvic cavity at the end of laparoscopic surgery should be performed to prevent this rare complication [8]. Morcellation in a containment bag is another method to reduce the risk of parasitic leiomyoma after laparoscopic power morcellation [4]. In addition, the retrospective analysis of Laganá et al. showed that posterior colpotomy and in-bag transvaginal extraction can be considered a feasible option for retrieval of surgical specimens after laparoscopic myomectomy [20]. We believe that for patients undergoing open surgery for uterine leiomyoma, a thorough inspection and washing of the abdominopelvic cavity at the end of the surgery is also vital.

## Conclusions

Clinically, parasitic leiomyoma should be considered in the differential diagnosis of a patient presenting with abdominal or pelvic solid tumors with a history of surgery for leiomyomas, even if the previous surgery did not involve the use of a power morcellator. In the process of transabdominal hysterectomy, tiny myoma tissues may also be produced and subsequently implanted into the surrounding tissues or organs, and then grow into parasitic myomas. Therefore, a thorough inspection and washing of the abdominopelvic cavity at the end of the surgery is necessary to ensure that no small tissue fragments remain, although this approach may not be sufficient to remove all myoma fragments. Further, patients should be informed of the risk of parasitic myomas in the future when making surgical plans.

## Data Availability

The data obtained during the current study are available from the corresponding author on reasonable request.

## Abbreviations

CT: Computed tomography
MRI: Magnetic resonance imaging
